# Development of a ^13^C Stable Isotope Assay for Dipeptidyl Peptidase-4 Enzyme Activity A New Breath Test for Dipeptidyl Peptidase Activity

**DOI:** 10.1038/s41598-019-41375-y

**Published:** 2019-03-20

**Authors:** Roger Yazbeck, Simone Jaenisch, Michelle Squire, Catherine A. Abbott, Emma Parkinson-Lawrence, Douglas A. Brooks, Ross N. Butler

**Affiliations:** 1 College of Medicine and Public Health, Flinders University, Adelaide, South Australia Australia; 2 Flinders Centre for Innovation in Cancer, Flinders University, Adelaide, South Australia Australia; 3 College of Science and Engineering, Flinders University, Adelaide, South Australia Australia; 4 School of Pharmacy and Medical Science, University of South Australia Cancer Research Institute, Adelaide, South Australia Australia; 5 School of Medicine, Trinity College Dublin, Dublin, Ireland

## Abstract

Dipeptidyl peptidase-4 inhibitors (DPP4i) are a class of orally available, small molecule inhibitors for the management of Type-II diabetes. A rapid, real-time, functional breath test for DPP4 enzyme activity could help to define DPP4i efficacy in patients that are refractory to treatment. We aimed to develop a selective, non-invasive, stable-isotope ^13^C-breath test for DPP4. *In vitro* experiments were performed using high (Caco-2) and low (HeLa) DPP4 expressing cells. DPP gene expression was determined in cell lines by qRT-PCR. A DPP4 selective ^13^C-tripeptide was added to cells in the presence and absence of the DPP4 inhibitor Sitagliptin. Gas samples were collected from the cell headspace and ^13^CO_2_ content quantified by isotope ratio mass spectrometry (IRMS). DPP4 was highly expressed in Caco-2 cells compared to HeLa cells and using the ^13^C-tripeptide, we detected a high ^13^CO_2_ signal from Caco2 cells. Addition of Sitaglitpin to Caco2 cells significantly inhibited this ^13^CO_2_ signal. ^13^C-assay DPP4 activity correlated positively with the enzyme activity detected using a colorimetric substrate. We have developed a selective, non-invasive, ^13^C-assay for DPP4 that could have broad translational applications in diabetes and gastrointestinal disease.

## Introduction

Dipeptidyl peptidase-4 inhibitors (DPP4i) are a class of orally available, small molecule inhibitors for the treatment and management of Type-II diabetes, which have been on the market for over 10 years. In 2006, Merck received approval from the FDA for their first in class DPP4i, Januvia® (Sitagliptin), and with several other inhibitors are referred to broadly as the ‘gliptins’ that include vildagliptin, saxagliptin and linagliptin^1,2^. Several meta-analyses have summarised the clinical efficacy of DPP4i, with almost all studies reporting overall improvements in the primary clinical endpoints of blood glucose, indicated by haemoglobin A_1C_, and bodyweight^3–5^. A sub-set of diabetic patients were classified as either poor or non-responders to DPP4i^6–8^, possibly suggesting differential DPP4 inhibition in non-responders. A rapid, real-time, functional test of DPP4 activity could help to define DPP4i efficacy in patients.

The current, gold standard for the quantification of DPP4 enzyme activity is the fluorometric or colorimetric enzyme assays^9,10^. However, these methodologies are cumbersome and are restricted to a single measurement from biological (blood and tissue) samples collected at a single point in time. Breath analysis represents a novel paradigm for the non-invasive detection and monitoring of health and disease^11–14^. Breath analysis has been widely used to detect functional pathophysiological changes^15–19^, and represents a novel method to quantify DPP4 enzyme activity.

**Figure 1:**
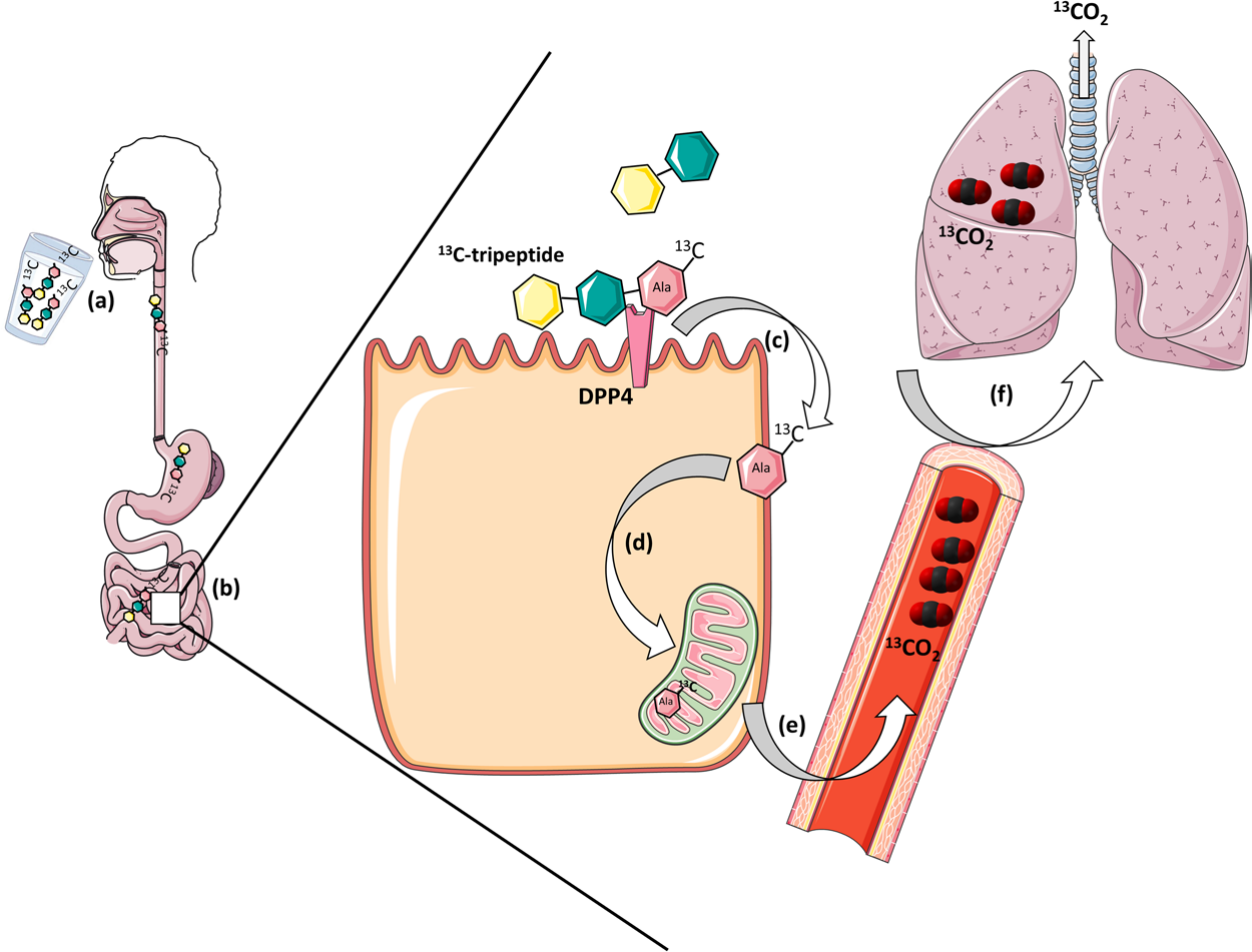
Schematic outlining the principle of a DPP4 breath test. (**a**) Following ingestion of the ^13^C-tripeptide in solution (water), (**b**) the ^13^C-tripeptide empties from the stomach into the duodenum where (**c**) it undergoes hydrolysis by DPP4, which is expressed on the epithelial cells of the intestinal brush border. (**d**) The liberated ^13^C-alanine is then absorbed by the cell where it is metabolised, leading to the formation of a ^13^CO_2_ bi-product, which (**e**) is transported via the blood (**e**,**f**) to the lungs where it is exhaled via the breath for collection and analysis by IRMS.

Stable isotope breath tests are primarily dependent on the ingestion of a specific isotopically labelled substrate, whose subsequent metabolism and incorporation into CO_2_ can be quantified in the exhaled breath (e.g. Fig. 1)^20^. ^13^C stable isotope breath tests have had the broadest clinical application to date, including tests for changes in liver function, exocrine pancreatic function, gastric emptying, and *Helicobacter pylori* infection^15–19^. There is an emerging recognition of the value of ^13^C-breath tests to rapidly and non-invasively predict and monitor response to pharmacological drugs.

Substrate design and delivery is critical for the specificity of any breath test. Ideally, a candidate non-invasive biomarker should possess unique functional characteristics that are altered when homeostasis is disturbed, such as altered metabolic pathways, differential enzyme expression, or a modified physiological state that ultimately leads to production of a CO_2_ bi-product, which is detectable on the breath^20^. DPP4 is a membrane bound serine protease with unique substrate specificity, cleaving dipeptides from the N-terminal of proteins with a penultimate proline or alanine residue^21,22^, and represents an excellent candidate for detection by ^13^C stable isotope breath testing (Fig. 1).

A stable isotope breath test for DPP4 enzyme activity could have broad research and clinical applications. As new DPP4i are developed, a non-invasive, functional breath-test could provide real-time information on inhibitor selectivity, pharmacokinetics and individual clinical response to DPP4i therapy. A DPP4 breath test could also have applications for defining disorders of immune regulation and altered signal transduction that are characterised by changes in DPP4 expression. The current study aimed to develop, *in vitro*, a ^13^C stable isotope enzyme assay that could detect DPP4 enzyme activity by isotope ratio mass spectrometry (IRMS). Furthermore, we aimed to determine assay specificity using the selective DPP4i, Sitagliptin, and how the assay correlated to the existing, gold-standard, colorimetric enzyme assay for DPP4.

## Results

### DPP4 is highly expressed in Caco-2 cells

To help confirm respectively high and low DPP4 expression in Caco-2 and HeLa cells, DPP gene expression was determined. DPP4 was highly expressed in Caco-2 cells, whilst very low gene expression was identified in HeLa cells (Table 1). Relatively high DPP8 gene expression was observed in both Caco-2 and HeLa cells (Table 1), but both DPP8 and DPP9 expression was lower in HeLa cells compared to Caco-2 cells. Relatively low expression of FAP was detected in HeLa cells, but there was little or no detectable FAP expression in Caco-2 cells.

**Table 1:** DPP gene expression in Caco-2 and HeLa cells.

	DPP4	DPP8	DPP9	FAP	PREP	TATA
Caco-2	13742.16 ± 444.37	4186.82 ± 143.22	707.51 ± 24.34	0.18 ± 0.17	5267.54 ± 254.25	6020.55 ± 178.49
HeLa	38.07 ± 1.58	1444.92 ± 127.32	106.71 ± 10.62	391.52 ± 42.90	1173.73 ± 72.79	3075.45 ± 153.69

Data is expressed as mean (n = 3 replicates) gene copy number/1 µg RNA ± SEM.

### ^13^C-alanine is metabolised *in vitro* by Caco-2 and HeLa cells

**Figure 2:**
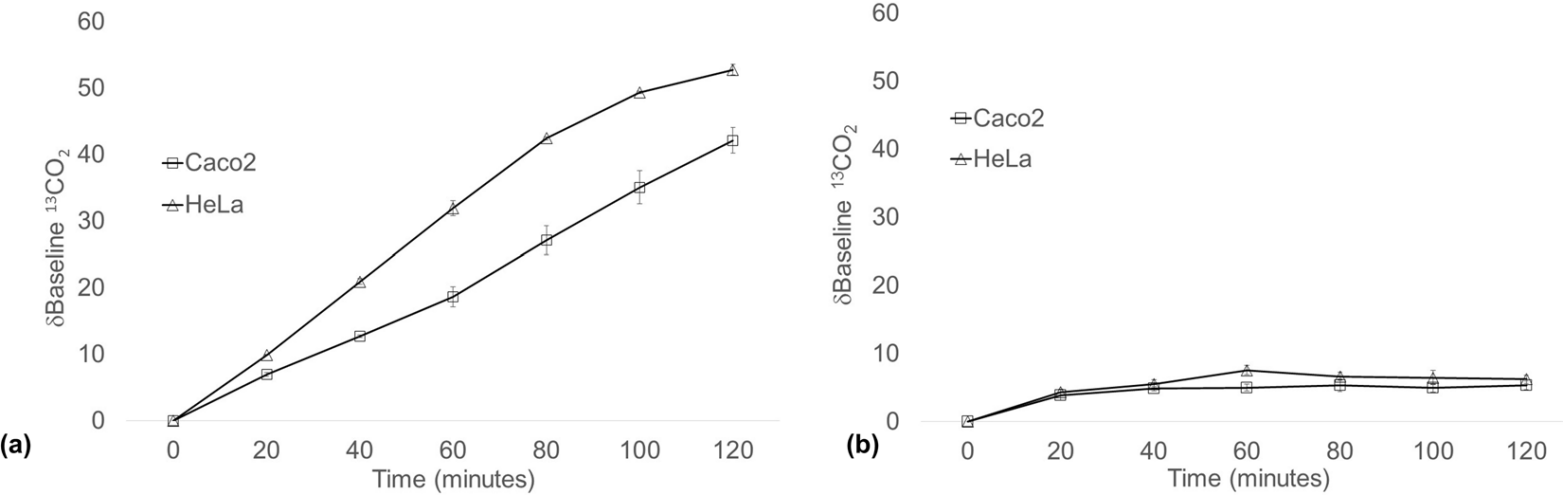
Headspace δbaseline ^13^CO_2_ of Caco-2 and HeLa cells after 2 h incubation with (**a**) ^13^C-glucose or (**b**) with media only. After baseline gas collection, cells received 2 mM ^13^C-glucose in fresh media. 10 mL gas samples were then collected from the cellular headspace over 2 h. Data is representative of a minimum of three separate experiments. Data expressed as mean ± SEM, n = 3 biological replicates.

The headspace sampling method was first optimised and validated in Caco-2 and HeLa cells using D-1-^13^C-glucose. Both Caco-2 and HeLa cells rapidly metabolised glucose, as indicated by an increasing δbaseline ^13^CO_2_ signal over the two-hour sampling protocol (Fig. 2a). The rate of increase in δbaseline ^13^CO_2_ was comparable between the two cell lines, but was higher in HeLa cells compared to Caco-2 cells over the sampling period (Fig. 2a). A background of 4–6 δbaseline ^13^CO_2_ signal was observed in all headspace samples taken from cells not receiving a ^13^C-substrate (Fig. 2b).

**Figure 3:**
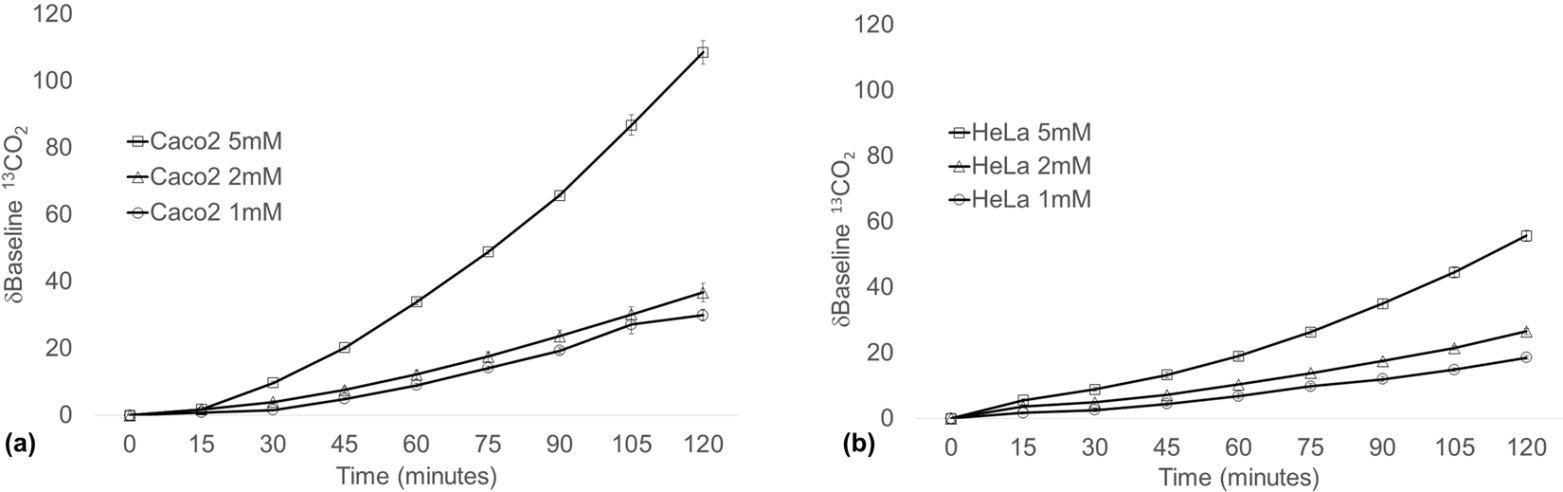
Headspace δbaseline ^13^CO_2_ of (**a**) Caco-2 and (**b**) HeLa cells after 2 h incubation with ^13^C-alanine. After baseline gas collection, cells received 1 mM, 2 mM or 5 mM ^13^C-alanine in fresh media. 10 ml gas samples were then collected from the cellular headspace over 2 h. Data is representative of a minimum of three separate experiments. Data expressed at mean ± SEM, in n = 3 flasks biological replicates.

^13^C-alanine metabolism was then determined in Caco-2 and HeLa cells. An increasing δbaseline ^13^CO_2_ was observed in both Caco-2 and HeLa cells over the 120 minute collection period for all ^13^C-alanine concentrations tested (Fig. 3). The δbaseline ^13^CO_2_ signal was highest in Caco-2 cells (108 ± 3.53; Fig. 3b) at the highest concentration of 5 mM ^13^C-alanine compared to HeLa cells (55.66 ± 1.72; Fig. 3a). However, the δbaseline ^13^CO_2_ was more comparable between cell lines at 1 mM and 2 mM ^13^C-alanine, with a δ^13^CO_2_ of 36.69 ± 2.83 and 26.55 ± 095 in Caco-2 and HeLa cells respectively at 2 mM ^13^C-alanine. This established the concentration of H-Gly-Pro-(^13^C_3_-Ala)-OH to be used in subsequent experiments.

### DPP4 activity can be detected *in vitro* by ^13^C-assay

**Figure 4:**
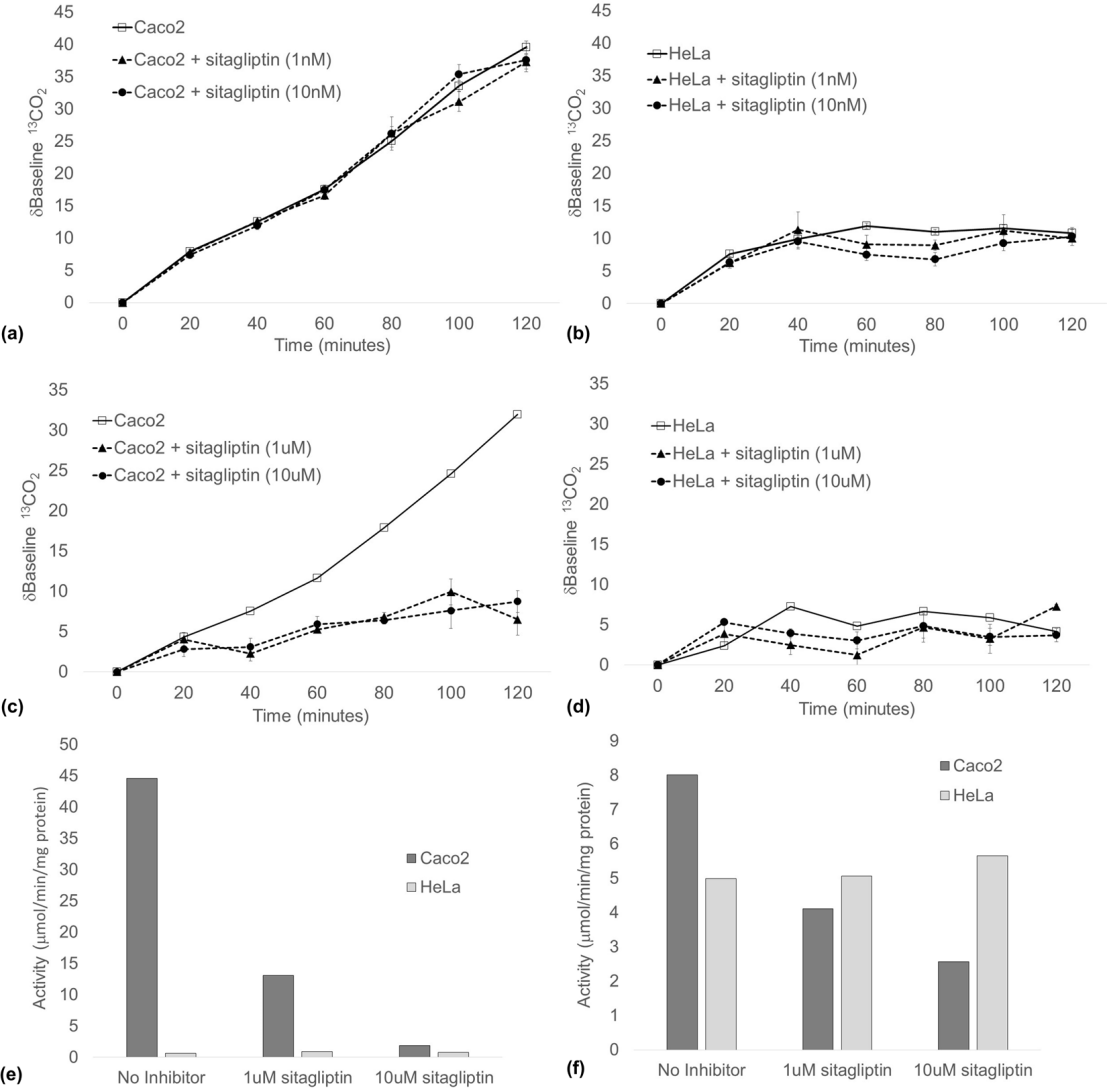
Headspace δbaseline ^13^CO_2_ in n = 3 flasks of (**a**,**c**) Caco-2 and (**b**,**d**) HeLa cells after 2 h incubation with H-Gly-Pro-(^13^C_3_-Ala)-OH. ^13^C-assay specificity was determined by addition of the selective DPP4i, Sitagliptin. After headspace collection was completed, cell extracts were collected, and DPP4 enzyme activity quantified in (**e**) membrane and (**f**) cytoplasmic fractions by the ‘gold-standard’ H-Gly-Pro-pNA colorimetric assay, with and without Sitagliptin. Data is representative of a minimum of three separate experiments. Data expressed at mean ± SEM.

The H-Gly-Pro-(^13^C_3_-Ala)-OH substrate was subsequently tested in Caco-2 and HeLa cells. An increasing δbaseline ^13^CO_2_ was observed in Caco-2 cells over the 120-minute collection period (Fig. 4a,c). In contrast, DPP activity, as indicated by the δbaseline ^13^CO_2_, was unchanged in HeLa cells, and was almost identical to the signal observed in non-H-Gly-Pro-(^13^C_3_-Ala)-OH controls (Fig. 4b,d).

The specificity of the ^13^C-assay for DPP4 was determined using the highly selective DPP4i, sitagliptin. δbaseline ^13^CO_2_ increased to a maximum of 39.58 ± 0.97 and 10.83 ± 1.18 in Caco-2 and HeLa cells respectively, and addition of 1 nM or 10 nM concentrations of sitagliptin made no change to the δbaseline ^13^CO_2_ signal (Fig. 4a,b). However, δbaseline ^13^CO_2_ was significantly reduced in Caco-2 cells treated with either 1 μM and 10 μM sitagliptin by up to 73%, compared to non-sitagliptin treated cells at 120 minutes (Fig. 4c). δbaseline ^13^CO_2_ was largely unchanged in HeLa cells over the collection period, and remained unchanged with the addition of either 1 μM or 10 μM sitagliptin (Fig. 4d).

DPP4 enzyme activity was then measured by the colorimetric assay in the corresponding Caco-2 and HeLa membrane extracts. Similar to the pattern of activity measured by ^13^C-assay, high DPP activity was observed in the Caco-2 membrane fraction (44.57 ng/min/mg protein) compared to low activity in HeLa cells (0.62 ng/min/mg protein; Fig. 4e). Addition of 1 μM or 10 μM sitagliptin to Caco-2 cell membrane extracts inhibited the DPP activity by 71% and 96% respectively, while there was no observable change in HeLa cells (Fig. 4e).

DPP enzyme activity was lower in Caco-2 cytoplasm compared to HeLa cells (Fig. 4f). Addition of 1 μM or 10 μM sitagliptin to the cytoplasmic fraction reduced the activity by 49% and 68% respectively (Fig. 4f). In contrast, DPP enzyme was higher in the HeLa cytoplasmic fraction (4.99 ng/min/mg protein) compared to the HeLa membrane fraction (0.62 ng/min/mg protein), and addition of 1 μM or 10 μM sitagliptin did not change the DPP activity.

**Figure 5:**
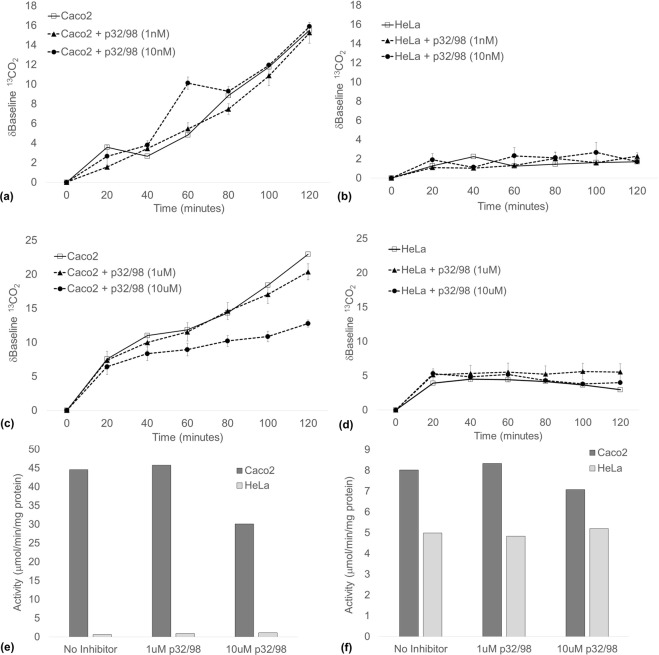
Headspace δbaseline ^13^CO_2_ in n = 3 flasks of (**a**,**c**) Caco-2 and (**b**,**d**) HeLa cells after 2 h incubation with H-Gly-Pro-(^13^C_3_-Ala)-OH. ^13^C-assay selectivity was determined by addition of the selective DPP4i, p32/98. After headspace collection was completed, cell extracts were collected, and DPP4 enzyme activity quantified in (**e**) membrane and (**f**) cytoplasmic fractions by the ‘gold-standard’ H-Gly-Pro-pNA colorimetric assay, with and without p32/98. Data is representative of a minimum of three separate experiments. Data expressed at mean ± SEM.

The selectivity of the H-Gly-Pro-(^13^C_3_-Ala)-OH substrate for DPP4 over other DPP enzymes was further defined using the less selective DPP inhibitor, p32/98 (Fig. 5). Nanomolar concentrations of p32/98 had no observable effect on enzyme activity measured by the ^13^C-assay (Fig. 5a,c). The δbaseline ^13^CO_2_ at 120 minutes was reduced by 44% only at the highest concentration (10 μM) of p32/98 in Caco-2 cells (Fig. 5c).

When measured by colorimetric assay, Caco-2 membrane DPP activity was similarly reduced at the highest concentration of p32/98 by 33% (Fig. 5e). Cytoplasmic DPP activity was again lower in Caco-2 cells compared to membrane activity; however, DPP activity was higher in the cytoplasmic fraction of HeLa cells compared to the respective membrane fraction (Fig. 5f). Addition of p32/98 did not significantly change the DPP activity in the cytoplasmic fractions of both cell lines (Fig. 5f).

### The ^13^C-assay positively correlates with the gold-standard assay

**Figure 6:**
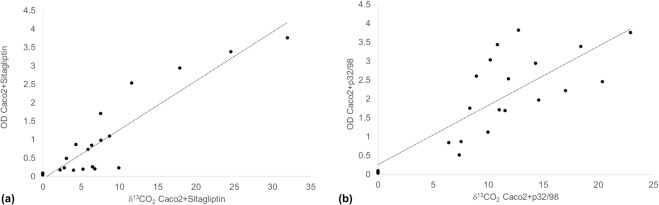
Pearson correlation between mean DPP4 activity measured by H-Gly-Pro-(^13^C_3_-Ala)-OH and by H-Gly-Pro-pNA colorimetric assay with the addition of either (**a**) sitagliptin (r = 0.91) or (**b**) p32/98 (r = 0.79), p < 0.0001.

To determine how well the ^13^C-assay correlated with the DPP4 gold standard, colorimetric assay, a Pearson correlation was performed between the two methods (Fig. 6). There was a strong correlation between activity measured by H-Gly-Pro-(^13^C_3_-Ala)-OH assay and colorimetric assay for both sets of experiments (r = 0.91 and r = 0.79 respectively, p < 0.0001).

## Discussion

To our knowledge, this is the first study to describe a non-invasive assay for the quantification of DPP4 enzyme activity in live cells using a ^13^C-labelled substrate. Using selective inhibitors of DPP4, we have demonstrated the specificity of the ^13^C-assay for DPP4 in live cell lines. The current study highlights the ^13^C assay for DPP4 as a useful tool for the non-invasive detection and monitoring of DPP4 activity in the presence and absence of DPP4i, with further *in vivo* pre-clinical and clinical studies being supported by the specific datasets presented herein.

DPP4i have emerged as an effective, orally available treatment modality for type-II diabetes^2,23^. However, a sub-set of patients are refractory to DPP4i, and individual variability between patients has been reported^6,8,24^. Previous studies have investigated predictive clinical parameters for the therapeutic benefits of DPP4i, including age and body mass^5^; however, to our knowledge, the level of DPP4 inhibition in individual diabetic patients treated with a DPP4i has not been reported. It is conceivable that differential systemic inhibition of DPP4 could result in differing glucoregulatory profiles. A ^13^C-DPP4 breath test represents a non-invasive, functional test of DPP4 activity that could be used for real time longitudinal quantification of responses to DPP4 inhibitors in type-II diabetic patients, potentially informing response to therapy and dose adjustment, negating the need for repeated blood draws. Furthermore, as new DPP4i continue to be developed, a DPP4 breath test represents a novel, non-invasive tool to investigate pre-clinical and clinical efficacy of new drug candidates. Pre-clinical studies are required to further validate the DPP4 breath test against a panel of clinically relevant DPP4i, comparing systemic and oral administration of the H-Gly-Pro-(^13^C_3_-Ala)-OH.

^13^C breath tests have had broad translational application, correlating to more invasive methodologies such as endoscopy and serology testing for *Helicobacter pylori* or gastric emptying by scintigraphy. Past studies have characterised DPP4 enzyme activity along the length of the gastrointestinal tract, reporting little to no activity in either the oesophagus or stomach. Preliminary findings in a human case study (data not shown) suggest that a DPP4 breath test may also have specific application for the non-invasive assessment of gut function and protein assimilation in individuals with suspected functional gastrointestinal disorders. In addition, DPP4 is highly upregulated in inflammatory conditions^9,23^ and this non-invasive test may provide an ideal method to monitor this immune pathogenesis in real time.

DPP4 is highly expressed along the brush border of the small-intestine, with an increasing gradient from the duodenum to the ileum^25,26^. Intestinal DPP4 has an important role in the hydrolysis of proline-rich proteins, such as those found within rye and barley that are otherwise resistant to enzymatic processing^27^. Intestinal brush border DPP4 has been previously implicated in the pathogenesis of coeliac disease^27,28^. More recently, Detel *et al*. reported a reduction in DPP4 activity of up to 74% in small intestinal biopsies derived from paediatric coeliac and malabsorption syndrome patients, also reporting a strong relationship between mucosal DPP4 activity and the degree of mucosal damage present in both patient groups^29^. Due to methodological limitations, DPP4 activity measurements have been limited to *ex vivo* assays in duodenal biopsies, meaning that longitudinal, integrated measurements of DPP4 activity have not been possible. A DPP4 breath test could establish a role for DPP4 in coeliac disease, and could be used as an early detection tool in paediatric patients. Alternatively, a ^13^C-DPP4 breath test could be used as a non-invasive, surrogate maker of intestinal integrity and brush border damage in coeliac disease, inflammatory bowel disease or intestinal mucositis.

The ^13^C-tripeptide was designed with a substrate specificity for DPP4, with metabolism of the liberated ^13^C-alanine leading to the subsequent production of a ^13^CO_2_ bi-product. Alanine is a non-essential amino acid that is converted to pyruvate before entry into the citric acid cycle, where it is completely oxidized to CO_2_^30^. Although alanine metabolism may be considered a rate-limiting step of the DPP4 breath test, our *in vitro* data indicated that both Caco2 and HeLa cells metabolised 2 mM alanine at a similar rate, so any observed changes were unlikely to be due to differences in alanine metabolism. This was further supported by a strong positive correlation between the ^13^C-DPP4 test and the gold-standard colorimetric assay. Validation of the breath test in human trials should first clearly define alanine metabolism in individuals using ^13^C-alanine to account for any possible variation.

A breath test for DPP4 enzyme activity must have high selectivity for DPP4 over other proteases in the DPP family. Sitagliptin is a small molecule, competitive binding inhibitor with high selectivity for DPP4 over other DPP family members^31^. Using sitagliptin, the ^13^CO_2_ signal was suppressed in Caco2 cells, suggesting the ^13^C-DPP4 assay was indeed detecting DPP4 enzyme activity. p32/98 is a less selective inhibitor for DPP4, also binding DPP8 and DPP9^32^, and the signal detected by the ^13^C-DPP4 assay was consistent with this, also correlating to the gold-standard colorimetric assay. The current findings suggest that the ^13^C-DPP4 assay is selective for DPP4. The emergence of the more specific DPP8/9 inhibitor^33,34^, 1G244, represents an opportunity to further test and validate the selectivity of the ^13^C-DPP4 assay.

The translational application and interpretation of a DPP4 breath test will largely depend on the mode of substrate delivery. For example, assessment of DPP4i response and efficacy in pre-clinical or clinical studies would likely seek to administer the Gly-Pro-Ala-^13^C via a sub-cutaneous route to measure systemic DPP4 activity. In contrast, the gradient of DPP4 activity along the gastrointestinal tract could be targeted using pH sensitive encapsulation techniques that specifically release the substrate in defined regions within the gastrointestinal lumen. Future iterations of the ^13^C-tripeptide may incorporate novel chemical design elements that specifically target intestinal vs systemic DPP4.

In conclusion, we have developed a ^13^C stable isotope assay that has selectivity to quantify DPP4 activity *in vitro*, and has a potential broad research and translational application in the development and assessment of new and existing DPP4i *in vivo*. Furthermore, the significant pool of DPP4 in the small bowel and in inflammatory conditions suggests that a DPP4 breath test could also have potential application as a non-invasive method to measure intestinal function/integrity and immune status. Certain cancers also exhibit high expression of DPP4 as exemplified by the adenocarcinoma cell line in this study and this may provide a measure of cancer activity and response to therapy.

## Methods

### Cell culture

All cell lines were purchased from CellBank Australia (New South Wales, Australia). Immortalised cell lines with high and low DPP4 expression and activity were selected for development of a ^13^C-DPP4 assay. Caco-2 cells are derived from a colorectal adenocarcinoma and possess a small-intestinal cell phenotype that includes high membrane DPP4 expression^35^. In contrast, HeLa cells are derived from cervical squamous cell carcinoma, and have very low to no membrane DPP4 expression or activity^36^.

Cells were grown in T75 tissue culture flasks under standard cell culture conditions in a 5% CO_2_, 37 °C incubator. Caco-2 cells were maintained in Dulbecco’s Modified Eagle’s Minimum Essential Media (DMEM) supplemented with 10% Foetal Bovine Serum (FBS), 1% sodium pyruvate, 1% non-essential amino acids and 20 mM L-glutamine. HeLa cells were grown in Eagle’s Minimum Essential Medium, supplemented with 10% foetal bovine serum and 20 mM L-glutamine.

### Absolute mRNA expression by qRT-PCR using external standards

RNA was extracted from Caco-2 and HeLa cells using the RNeasy Kit (Qiagen, Germany) combined with the Qia-shredder (Qiagen, Germany) in accordance with manufacturer’s instructions. Complementary DNA (cDNA) was synthesised from 1 µg of RNA using the Quantitect Reverse Transcription Kit (Qiagen, Germany). 2.5 ng of cDNA was analysed using qPCR in real-time on the Rotor Gene Q (Qiagen, Germany). 10 µL qPCR reactions consisted of 100 nM primer, Kapa SYBR Fast qPCR Universal Mastermix (Kapa Biosystems, USA). Primer sequences are detailed in Table 2. Cycling conditions were as follows: 1 cycle at 95 °C for 3 minutes for enzyme activation, followed by 45 cycles of denaturation at 95 °C for 3 seconds and Annealing/Extension/Data Acquisition performed at 68 °C for 30 seconds. External PCR standards for each gene were included in each run. These consist of purified PCR products of known gene copy number prepared using previously published methods^37^. All samples and standards were analysed in triplicate. Data was expressed as a ratio of the number of copies of the gene of interest to the number of copies of the tata box protein (TATA). Error bars represent standard deviation of samples performed in triplicate.

**Table 2:** Primer sequences for quantitative real-time PCR reactions.

Gene	Primer Sequences (5′-3′)	Product length (bp)
DPP4	F: ACG CCG ACG ATG AAG ACA CCG	95
	R: TTC AGC AGA ACC ACG GGC ACG	
DPP8	F: ACA TGA TGG CTA AGG CAC CAC ATG A	106
	R: CTG TTC TCA CCA GAC ATG GCA AGG	
DPP9	F: TTGCTCTGACCAGCGGTGAATG	142
	R: GCCTCATAGCTGACCACGTAGA	
FAP	F: ATG GGG CTG GTC CTA TGG AGG AT	97
	R: GCT GGA GAC TGG AGC CAC TGC	
TATA	F: CGA AAC GCC GAA TAT AAT CCC AAG CG	137
	R: GCC AGT CTG GAC TGT TCT TCA CTC TT	

F = Forward and R = Reverse.

### Design and optimisation of gas sampling method

Gas sampling lids were custom designed for all gas collection experiments. Briefly, the lid of a solid (non-vented) T75 tissue culture flask was pierced with an 18-gauge needle, and silicone gel was then used to seal the area around the needle and secure its position. A two-way tap was attached to the needle to facilitate gas collection by 10 mL syringe^38^.

As all mammalian cells actively metabolise glucose, D-1-^13^C-glucose (Cambridge Isotopes, USA) was used to optimise and validate the headspace collection method. Briefly, flasks (n = 3/cell line) containing confluent Caco-2 or HeLa cells were incubated for 20 minutes at 37 °C in a 5% CO_2_ incubator, and a baseline (t = 0) 10 mL gas sample was collected into 12 mL evacuated vacutainers (Exetainer, USA). 10 mL of room air was then injected into the cellular headspace to replace the sample volume of gas^38^. The original cell media was then discarded and replaced with 2 mM D-1-^13^C-glucose dissolved in 3 mL of fresh cell media.

Gas sampling lids were then re-attached and flasks returned to the 5% CO_2_ incubator. Gas samples were collected every 20 minutes for a total of 120 minutes. ^13^CO_2_ was quantified in gas samples by ABCA isotope ratio mass spectrometry (IRMS) (Europa Scientific, Sercon, Cheshire, UK). Concurrent with every experiment, headspace samples were collected from cells incubated with media only.

### Design and optimisation of DPP4 selective ^13^C-tripeptide

Alanine is a non-essential amino acid whose metabolism via the glucose-alanine cycle leads to the production of a CO_2_ bi-product. ^13^C-alanine (Isotec, USA) was selected as the reporter moiety of the ^13^C-tripeptide. To confirm that there were no differences between Caco-2 and HeLa cells in alanine catabolism, Caco-2 and HeLa cells were incubated with 1 mM, 2 mM or 5 mM ^13^C-alanine, as previously described, and headspace samples collected every 20 minutes for a total of 120 minutes.

The ^13^C-tripeptide was subsequently designed with a proline in the penultimate position, to confer selectivity for DPP4. The full tripeptide sequence was H-Gly-Pro-(^13^C_3_-Ala)-OH and was synthesised by Cambridge Isotopes (USA). 3 mM H-Gly-Pro-(^13^C_3_-Ala)-OH dissolved in 3 mL of media was then added to confluent flasks of Caco-2 and HeLa cells and headspace samples collected every 20 minutes, for 120 minutes as previously described.

### H-Gly-Pro-(^13^C_3_-Ala)-OH assay selectivity

Assay selectivity was determined using the selective inhibitors of DPP4 and DPP enzyme activity sitagliptin phosphate monohydrate (Biovision, USA, #1757)^32^ and p32/98 (Enzo Life Sciences, USA, #BML-PI142-0050)^32^. Caco-2 and HeLa cells were treated with 1 nM, 10 nM, 1 μM and 10 μM sitagliptin or p32/98 for 5 hours prior to headspace analysis. After collection of the baseline headspace sample (t = 0), the media was discarded and replaced with 3 mL of fresh media containing 3 mM H-Gly-Pro-(^13^C_3_-Ala)-OH and the corresponding concentration of sitagliptin or p32/98.

### Gas sample analysis

^13^CO_2_ was quantified in headspace samples by ABCA IRMS (Europa Scientific, Sercon, Cheshire, UK), which measured the ratio of ^12^C:^13^C. 5% CO_2_ reference gas was used to calibrate the IRMS prior to sample analysis. In addition, throughout the sample run, reference and quality control gas (High Purity Carbon Dioxide; BOC, Australia) was analysed every 10–20 samples to minimise drift. All samples were analysed within 24–48 hours of sample collection. All data was expressed as the mean δbaseline ^13^CO_2_ of n = 3 replicates per cell line per experiment ± standard error of the mean and is representative of at least two experimental repeats.

### DPP enzyme activity assays

Flasks of confluent cells were rinsed with 7 mL of cold phosphate buffered saline (PBS) then scraped into 10 mL of PBS and centrifuged at 15,000 G for 10 minutes at 4 °C. Cell pellets were resuspended in 1–1.5 mL of phosphate buffered saline with protease inhibitors, and extracts separated using the ‘freeze-thaw’ method. Samples were again centrifuged for ten minutes at 15,000G at 4 °C. The supernatant (representing the cytoplasmic fraction) was then removed and stored at −80 °C. The remaining pellet was resuspended in 200 µL of PBS with 1% TritonX-100 and then centrifuged. The supernatant (representing the membrane fraction) was aspirated and stored at −80 °C until further analysis.

DPP enzyme activity was measured in cell cytoplasmic and membrane fractions using a colorimetric enzyme assay, modified from the original method described by Hopsu Havu *et al*.^39^. The colorimetric DPP substrate, H-Gly-Pro-p-nitroalinilide (pNA; extinction coefficient: 9450 M^−1^ cm^−1^) (Bachem, Switzerland, #L-1100) was used at a final concentration of 1 mM in all assays. Dual absorbance readings were taken at 405 nm and 600 nm, every 10 minutes at 37 °C for a total of 90 minutes, using a ClarioStar microplate reader (BMG Labtech, Germany). DPP8/9 activity was determined by addition of the selective DPP4i, Sitagliptin and the non-selective inhibitor p32/98, at concentrations of 1 nM, 10 nM, 1 μM and 10 μM.

Protein content was determined in 10 μL of sample using a BioRad Detergent Compatible (DC) Assay kit with Bovine Serum Albumin (BSA) standards. Final enzyme activity was expressed as μmol/min/mg of protein.

### Statistics

To determine the relationship between the H-Gly-Pro-(^13^C_3_-Ala)-OH stable-isotope assay and the H-Gly-Pro-pNA colorimetric assay, a Pearson correlation was performed using IBM SPSS Statistics V23 between δbaseline ^13^CO_2_ and raw optical density readings. p < 0.05 was considered a statistically significant relationship.
